# The impact of motivational interviewing on relapse to substance use among women in Iran: a randomized clinical trial

**DOI:** 10.1186/s12888-020-02561-9

**Published:** 2020-04-10

**Authors:** Sonia Oveisi, L. A. R. Stein, Elham Babaeepour, Marzieh Araban

**Affiliations:** 1 Department of Pediatrics, Faculty of Medicine, Metabolic Disease Research Center, Qazvin University of Medical Sciences, Qazvin, Iran; 2 Psychology Department, University of RI, Kingston, RI USA; 3 Behavioral & Social Sciences Department, Brown University School of Public Health, Providence, RI USA; 4 RI Training School, Cranston, RI USA; 5 Center for Prisoner Health & Human Rights, Brown University Medical School, Providence, RI USA; 6 Department of Midwifery, Faculty of Nursing and Midwifery, Qazvin University of Medical Sciences, Qazvin, Iran; 7 Department of Health Education and Promotion, Public Health School, Ahvaz Jundishapur University of Medical Sciences, Ahvaz, Iran

**Keywords:** Motivational interviewing, Female drug user, Relapse, Stages of change model, Addiction

## Abstract

**Background:**

Women in Iran are in great need of effective substance abuse services. The current study was conducted to investigate the effectiveness of motivational interviewing (MI) for women in treatment for drug use in Iran.

**Method:**

The sample (*N* = 60) included women in a drug treatment center in Qazvin (Iran) from August to December of 2017. The research sample included 60 female drug users randomly assigned to MI or Standard Care (SC). Prior to randomization women completed a baseline questionnaire and the Relapse Prediction Scale (RPS), which measures desire (urge) to use and probability of using/not using in risky situations (self-efficacy). MI consisted of eight 60-min group sessions over a 1-month period, twice weekly. At 2-months follow-up, data were gathered using a questionnaire similar to baseline. Mixed Model Analysis were used to determine group differences.

**Results:**

Mean age of participants was 30 years and average addiction duration was 7 years. Although the scores of the desire to use and the probability of drug use were not significant before the intervention, after the intervention, scores on desire to use and probability of use improved about 81.1% (F: 2230.15, *P* < 0.001, degrees of freedom: 63, 15) and 81.9%, (F: 749.39, P < 0.001, degrees of freedom: 79, 77), respectively, compared to those of control group.

**Conclusion:**

The results showed that motivational interviewing could decrease desire to use and probability of use among female drug users. Motivational interviewing could play an important role in improving women’s health in Iran.

**Trial registration:**

IRCT registration number: IRCT20140907019077N4

Registration date: 2017-12-12, 1396/09/21

Registration timing: registered_while_recruiting

Last update: 2017-12-12, 1396/09/21

## Background

### Women and drug abuse

Given Iran’s proximity to a major opioid trade route, illicit drug use is a major problem in Iran and opioids in particular are readily available [1–4]. Due to stigma, prevalence research on drug use is scant, although use of stimulants and injection drugs appears to have increased over time.

Amin-Esmaeli et al., (2016) [4] studied prevalence of substance use disorders in Iran (*N* = 7841; N, women = 4475). Prevalence of 12-month use disorder for any drug was 2.44% with opioid use disorder most common (2.23%) followed by cannabis (.56%) and amphetamines (.39%). Substance disorder was more likely in men, divorced persons and persons of lower socio-economic status; and over half of the sample had unmet treatment need. Very few women use substance services and this is likely due to lack of facilities specializing in women, cost and stigma [4]. Other studies have also found relatively few women seeking substance treatment [2] .

A national survey of emergency hospitals in Iran indicated 2% of women used opium with .05% dependent on opium and its derivatives [5]. In another national survey in Iran, about 3.3% of women ages 15 to 64 years had a life-time history of narcotics and/or stimulants use [5]. There is a high prevalence of stimulant use, such as crystal methamphetamine, in Iran [5]. In a sample of women in substance treatment, use of crystal meth appears to be associated with being single and an expectancy that it enhances the ability to work, whereas use of opium is associated with being married and having a spouse who is drug-involved [5].

Pre-treatment characteristics of persons in substance treatment in Iran were examined and only 4% (*N* = 33) of the sample was female [1]. Of these women, 63% were homemakers, 78% had a high school degree or more, 70% were married, 33% were 25–34 years old, and main drugs of abuse were opium (59%), crystalline heroin (heroin hydrochloride, 34%) and other drugs (6%). Ghaderi et al. (2017) studied gender differences in drug use between Iranian men and women receiving methadone maintenance treatment (MMT) and found methamphetamine use and simultaneous use of multiple substances during the last 12 months were less common among women. Similarly, life-time dependence on nicotine, heroin and alcohol; and life-time cannabis and other substance abuse were less common among women. Among women, the most frequent 12-month substance diagnosis was opium dependence (42.5%), whereas the most frequent life-time substance diagnosis (aside from opioid) was nicotine dependence.

Aside from opioids, 12-month prevalence rates for common illegally used substances in Iran are as follows: Alcohol, 2%; cannabis, 1%; and methamphetamine, .5% [3]. Substance use and dependence are increasing among women in Iran and therefore, clinics specializing in the addictions for women are opening [3]. Although women use substances at lower rates than men, women who are in need do not get needed and critical intervention. Therefore, when women do seek treatment, it is important that they receive intervention that is useful in assisting with change in drug use.

### The trans-theoretical model (TTM) and motivational interviewing (MI)

The TTM is one of the most frequently used and tested models of behavior change [6]. It provides a foundation for tailoring interventions based on readiness to change, the central organizing construct of the TTM [7]. Readiness is characterized in terms of five stages: Pre-Contemplation (no intention to change in the next 6 mo); Contemplation (intention to change within 6 mo); Preparation (intends to take action within 30 days and has a plan); Action (behavior change has occurred); and Maintenance (change sustained for > 6 mo; see Ha et al., 2003). Other core constructs include decisional balance, self-efficacy, and the processes of change [7]. These constructs have been validated with many behaviors across a variety of populations [8]. The TTM has been useful in designing interventions because it accounts for readiness to change in tailoring interventions [9].

MI provides an empirically supported style for matching counseling to an individual’s readiness to change [10].MI represents a practical approach for behavior change by enhancing a client’s own internally motivated change process, and dovetails well with other behavioral interventions including the TTM [11]. Responsibility for behavior change is assumed to lie within the individual, and ambivalence is recognized as a natural part of this change process. MI is designed to assist clients in working through ambivalence and in moving toward change. The MI counselor uses techniques including personalized feedback, reflective listening, exploring pros/cons of change, supporting client self-efficacy, eliciting “self-motivational statements” (problem recognition, intention to change, optimism about change), and generating solutions to potential change barriers. Of critical importance, MI emphasizes the client’s personal choice regarding change, de-emphasizes labeling the client and his/her behaviors, and avoids arguing with or confronting the client with the need to change. Meta-analytic work has found that MI is efficacious across a variety of settings, for a wide range of health behaviors including substance use, risky sex and treatment engagement [12]; appears particularly useful for minority populations, at least in the United States [13]; and can be effective in as little as one session [14, 15]**.**

### Study rationale

Women do not access drug treatment when in need, therefore it is important to provide them with useful intervention when they do enter treatment. A large literature base supports use of TTM and MI in making behavior change with respect to substance use, and accounts for cognitive factors during the process of behavior change, including dealing with tempting situations and developing efficacy to avoid substance use. This study evaluates the use of MI that is informed by TTM to address women’s reported ability to cope with tempting situations. It is important because more work needs to be done to assist women in Iran, use of TTM and MI should be better evaluated for use outside of Western countries, and this study stands to lay the foundation to conduct future research on actual behavior change.

## Methodology

The focus of this trial was to study substance use problems, including opium use in particular. However, treatment of poly-substance use was identified as a much greater need during implementation. Therefore, this paper focuses on treatment of persons with a wide-range of substance use disorders. Original planned outcomes included relapse to substance use and results of the Relapse Prediction Scale (RPS; see below). As the planned 2-month follow-up makes it difficult to observe relapse, this study focuses on the RPS, which is a more sensitive and realistic outcome, given the small sample size and brief follow-up.

### Sample

It was a parallel trial with a 1 to 1 ratio. The sample (*N* = 60) consisted of women ages 15–49 years old receiving substance intervention at a treatment site in Qazvin (Iran) in 2017. The inclusion criteria were being fluent in Farsi (the Iranian official language), not having any chronic physical health disorder and not being pregnant. Healthy volunteers were accepted. A researcher approached women to ask if they might be interested in volunteering for a confidential intervention study. Procedures were explained and women could opt out anytime. Written informed consent was obtained. Women received a small gift for completing questionnaires.

### Measurements

Basic demographic data were collected on women, including age, substance use and so forth. To examine the risk to return to substance use, the Relapse Prediction Scale (RPS) was used. The RPS consistes of 45 items (Wright, Beck, Newman & Liese, 1993) [16]. Each item consists of a situation where the respondent rates strength of urge to use and likelihood of use.

For example, it is expressed as the following questions, where each item is rated for urge and probability, separately:

1. I am in a place where I have used drugs before.
2. I am with the people whom I have used drugs with.
3. I see my husband who uses drugs.

All the questions are graded on a 5-point Likert type scale consisting of 0-none, 1-poor, 2-moderate, 3- strong, 4- very strong. Scores rage from 0 to 180 for urge and likelihood of use, separately, with higher scores representing more risk. Prior studies of the Farsi/Persian-translated version indicate internal consistencies ranging from .74 to .81 [17, 18]. Gholami & Shareh (2015) [3] found that the scales distinguished between treatment and control groups as expected in substance-dependent persons (e.g., craving and likelihood of drug use was reduced), supporting scale validity. In the current research, Cronbach’s alpha was found to be 0.91.

### Implementation method

At baseline, women completed an assessment, including the RPS via interview with a researcher blind to study condition. The sample then was randomly assigned to experimental intervention and standard care (SC). Randomization was achieved using sealed, opaque, sequentially numbered envelopes developed from a random number generator. A research assistant who was not involved in the recruitment of participants prepared the envelopes. Experimental intervention consisted of group-based MI sessions held for eight 60-min sessions (see Table 1). Sessions occurred over a 1-month period, twice weekly. SC included 4 sessions of individual counseling in which women were encouraged to seek outside support, including self-help, which is the most commonly used substance intervention in Iran [4] . These sessions occurred at once per week. At 2-months follow-up, data were gathered using a questionnaire similar to baseline questionnaire. Women were in the program 10–14 days before interventions began. At follow-up they were no longer in the treatment facility. Baseline and follow-up assessments were conducted by research staff and not intervention providers for both treatment groups.

**Table 1:** The structure and contents of motivational interviewing sessions

Session	Content
**First**	Introduction: Norms and procedures of group, introducing motivational interviewing and stages of change, determining stage of change.
**Second**	Describing a typical day: Describing substance use in terms of quantity, physiological effects, signs of substance problems. How to monitor substance use with a screening log.
**Third**	Expectations: Discussing what we think substances do for us, why we take them, the good and not so good of use.
**Fourth**	Self-efficacy and temptation: Recognizing triggers and tempting situations; comparing tempting situations and confidence to use or not in these situations.
**Fifth**	Rewarding successes: How to recognize successes, setting goals and then rewarding yourself.
**Sixth**	Efficacy: Practicing refusal of drugs using role plays.
**Seventh**	Urge: Dealing with urges, how to avoid them and cope with them, identifying other enjoyable activities, alternatives to substance use.
**Eighth**	Slips: Using a slip to learn, reviewing past reasons for changing use, resources available and what can be done after a slip; summary and conclusion.

### MI sessions

MI sessions were based on, “Group treatment for substance abuse: A stages-of-change therapy manual”, which has been translated to Farsi [19]. Table 1 presents structure and content of sessions. Consistent with MI, sessions incorporated affirmations, open questions, reflections and selective summary to ellicit desire, ability, reason, need and commitment to change [20]. Interventionists met women where they were in their desire to change (e.g., precontemplation, making changes, etc); utilized decisional balance (pros/cons of change) to enhance interest in change; assisted women to enhance self-efficacy for change (“what about you makes you think you could make a change if you decided?”); and examined tempting situations to assist them in problem-solving risky situations. These techniques are reflected in the TTM and are consistent with MI.

Providers led the sessions for attendees, and those who offer MI sessions did not offer standard care drug treatment sessions. Providers had 30 h of training using materials in Miller & Rollnick (2013) and Prochaska et al. [9, 21]. Training was conducted by EB. Supervision occurred every other week for the duration of the study and included role-plays with feedback and case discussion.

### Standard care (SC) treatment

SC generally did not involve medication assisted treatment for substance use. SC included weekly doctor visits and twice a week sessions guided by a non-profit organization, “Addicts Anonymous”. In addition, 4 sessions were delivered by a psychologist covering general psycho-education on drugs, and basic behavioral techniques including drug-refusal skills [22].

### Statistical analysis

Statistical Package for the Social Sciences-version 22 (SPSS Inc., Chicago, IL, USA) was used to conduct descriptive and inferential statistics. Differences in sociodemographic characteristics between groups were assessed using a t-test and chi-squared. At first all data were checked for normality using Kolmogrov-Sminov test; data met normality assumptions. Mean differences between intervention groups were compared in repeated measures analysis. In particular, mixed model repeated measures analyses were used to determine the effect of intervention on urge (desire to use) and probability of use (or efficacy). A *p*-value of < 0.05 was considered statistically significant.

Considering the mean and standard deviation of relapse from a previous study [23], using the below formulla with *α* = 0.05 and *β* = 0.1, a sample of 30 was considered satisfactory for each group.

$$ n=\frac{{\left({z}_{1-\frac{a}{2}}+{z}_{1-B}\right)}^2{\sigma}^2}{{\left({\mu}_1-{\mu}_2\right)}^2} $$

### Ethics

The study was approved by the Ethics Committee of Qazvin University of Medical Sciences (IR.QUMS.REC.1396120). All participant asked to give informed consent.

## Results

**Fig. 1:**
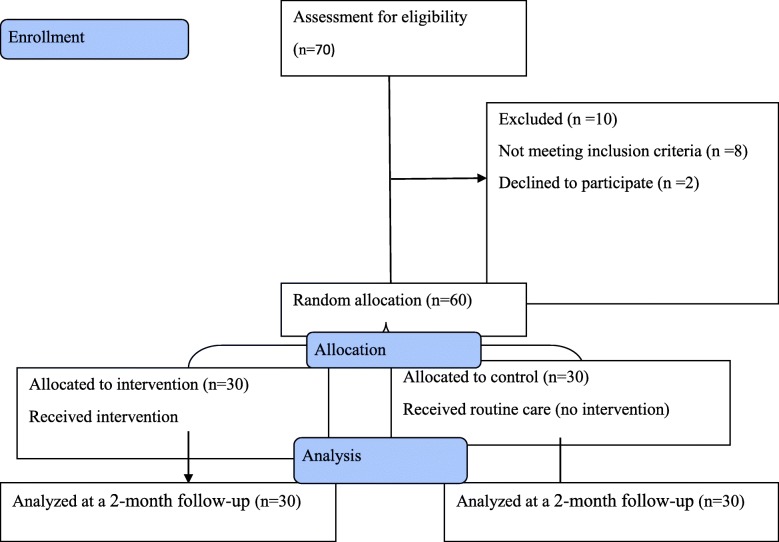
Flow diagram of the study participants

The average age of participants was 30 years old and both groups were not significantly different in terms of education and income *P* > 0.05. Figure 1 shows flow diagram of the study. The highest rate of substance use was associated with methamphetamine and heroin in both groups (see Table 2 for description of other drugs). An average duration of addiction was reported as 7 years. Also, women reported seeking treatment 2–3 times previously. Both groups had relatives who were drug users, particularly their husbands. In brief, both groups were not significantly different in terms of demographics characteristics, P > 0.05. (See Table 3.)

**Table 2:** Drug Use Rates by Treatment Group

	Experimental group								Control group							
	Daily		Weekly		Monthly		Irregularly		Daily		Weekly		Monthly		Irregularly	
	N	%	N	%	N	%	N	%	N	%	N	%	N	%	N	%
**Other drugs**	19	63	3	10	2	6.6	6	20	20	66.6	2	6.6	0	0	8	26.6
**Heroin**	23	76	2	6.6	2	6.6	5	16.6	18	60	2	6.6	0	0	10	33.3
**Opium**	15	50	10	33.3	4	13.3	1	3.3	16	53.3	4	13.3	2	6.6	6	20
**Meth-amphet-amines**	28	93	0	0	0	0	2	6.6	29	96.6	0	0	0	0	1	3.3

**Table 3:** Demographic properties of the study population at baseline

		***Intervention*** ***n = 30***	***Control*** ***n = 30***	
***Variables***		Mean (SD)	Mean (SD)	*P*_Value_
***Age***		30.93 (7.47)	30.90 (7.76)	0.9^a^
***Years of addiction***		6.90 (7.83)	6.49 (7.37)	0.7^a^
		N (%)	N (%)	
***Income***	poor	15 (50)	14 (46.6)	0.1^b^
	Above moderate	15 (50)	16 (53.4)	
***Education***	Lower than high school diploma	21 (70)	21 (70)	0.5^b^
	Higher than High school diploma	9 (30)	9 (30)	
***Rehabilitation experience***	Yes	23 (76.7)	18 (60)	0.1^b^
	No	7 (23.3)	12 (40)	
***Addiction among family members***	Yes	26 (86.7)	25 (83.3)	0.7^b^
	No	4 (13.3)	5 (16.7)	

• ^a^Using t test

•^b^sing chi-square test

The descriptive statistics of the RPS are provided in the Table 4. As observed in the table, the results reflect the effectiveness of MI (*P* > 0.001).

**Table 4:** Comparisons of the urge and likelihood of substance abuse between two groups using Mixed Model Analysis

	Before intervention		After intervention		F	DF	P
	Intervention (n = 30)	Control (n = 30)	Intervention (n = 30)	Control (n = 30)			
	Mean (SD)	Mean (SD)	Mean (SD)	Mean (SD)			
**Probability of substance abuse**	3.33 (0.67)	3.27 (0.34)	0.60 (0.41)	2.91 (0.32)	**2230.15**	**63, 15**	**0.001**
**Desire for substance abuse**	3.17 (0.60)	3.52 (1.06)	0.58 (0.23)	3.05 (0.41)	**749.39**	**79, 77**	**0.001**

## Discussion

As compared to women randomized to standard care (SC), women randomized to motivational interviewing (MI) significantly reduced both desire (urge) to use substances and reported probability of using in tempting situations (i.e., self-efficacy improved). Results are consistent with prior research in the filed [24].

Addiction is often a relapsing condition (most of the women had been in treatment multiple times) and is associated with much stigma and ambivalence around change. Therefore, MI may be particularly useful in reducing substance abuse in that it meets women where they are in change (or cycling through stages of change), assists women to resolve ambivalence, and is non-judgmental and person-centered [20].

### Limitation

Results may not generalize to addicted women in other settings since the samples were recruited from one rehabilitation center. Women were in a treatment program when they responded, which might have biased their responses. Also, there was no behavioral outcome included such as actual drug use following release from the facility. In addition, differences in treatment exposure (MI and SC had 8 and 4 sessions, respectively) could account for group differences. Also we did not assess family support, which may impact relapse.

## Conclusion

Results showed that motivational interviewing can decrease desire (urge) to use and reported probability of use (i.e., improve self-efficacy) among female drug users. Motivational interviewing could play an important role in improving women’s health in Iran, although it is not regularly used currently in Iran. This study is a critical first step at adapting and evaluating MI in Iranian women to reduce substance use. It is important to evaluate MI and TTM-informed interventions in non-Western countries. Findings are encouraging and future work should evaluate behavioral outcomes in larger samples.

## Data Availability

The datasets generated and/or analyzed during the current study are not publicly available due to cost to prepare materials for public repository, but they are available from the corresponding author upon reasonable request.

## Abbreviation

RPS: Relapse Prediction Scale
